# From barriers to benefits: A personalized sleep intervention enhances sleep duration and emotional health in chronic short sleepers

**DOI:** 10.1111/bjop.70075

**Published:** 2026-04-18

**Authors:** Wei Wang, Ho Yin Derek Ma, Ming Ming Chiu, Shu Fai Cheung, C. Harry Hui, Mark Lawrence Wong, Janet Hui‐wen Hsiao, Rong Wei Sun, Yeuk Ching Lam, Akira Basa Okabe, Chin Ho Chan, Esther Yuet Ying Lau

**Affiliations:** ^1^ Department of Psychology The Education University of Hong Kong Hong Kong China; ^2^ Analytics\Assessment Research Centre The Education University of Hong Kong Hong Kong China; ^3^ Special Education and Counseling Department The Education University of Hong Kong Hong Kong China; ^4^ Department of Psychology University of Macau Macau China; ^5^ Department of Psychology The University of Hong Kong Hong Kong China; ^6^ Department of Social and Behavioral Sciences City University of Hong Kong Hong Kong China; ^7^ Division of Social Science Hong Kong University of Science and Technology Hong Kong China; ^8^ School of Arts and Humanities Tung Wah College Hong Kong China; ^9^ Clinical Health Psychology Services North East London NHS Foundation Trust London UK; ^10^ Academy for Applied Policy Studies and Education Future, Centre for Psychosocial Health The Education University of Hong Kong Hong Kong China

**Keywords:** ecological momentary assessment, emotional functioning, insufficient sleep, mixed‐methods research, sleep barriers, sleep behaviour change

## Abstract

This pilot study evaluated a personalized sleep intervention incorporating motivational interviewing techniques to address sleep barriers, along with tailored sleep hygiene and extension for chronic short sleepers. Eleven university students completed a 14‐day sequential intervention (baseline, sleep hygiene and a combined phase adding 90‐min extension), assessed via actigraphy, daily diaries, ecological momentary assessments and qualitative interviews. The intervention was highly feasible. Total sleep time increased from 5.01 h (baseline) to 5.62 h (sleep hygiene) and 6.67 h (combined phase), alongside reduced bedtime procrastination and improved sleep hygiene practices. Multilevel modelling suggested that sleep hygiene increased time in bed (+0.72 h) and morning vitality, while sleep extension further extended time in bed (+1.49 h) and total sleep time (+0.55 h). Emotional benefits were linked to within‐person increases in sleep duration. Qualitative findings highlighted heterogeneous barriers and the importance of context‐based personalization. These preliminary results support the potential of personalized sleep interventions to improve sleep and emotional health in chronic short sleepers, warranting further controlled trials.

## BACKGROUND

Sleep insufficiency and instability are prevalent; even among individuals meeting the recommended 7–9 h of sleep, 28% of nights fall short, and only 2% maintain stable adherence (≥6 nights/week) (Scott et al., 2024). These short and fluctuating sleep duration trajectories are associated with significantly higher risks of adverse physical, emotional, and neurocognitive outcomes (Wang et al., 2025). The efficacy of sleep interventions to improve sleep health across diverse populations has gained increasing attention. Among healthy adults, behavioural interventions are the most widely used and effective, whereas sleep hygiene (SH) could improve knowledge and sleep change intention but may fail to translate into sustained sleep extension (SE) behavioural changes (Spiller, 2025). Combining education with behavioural strategies shows promise but remains understudied (Gadam et al., 2023). Recent feasibility studies have examined the specific components of successful extension programs, such as combining educational emails with wearable technology and supportive coaching to increase accountability (Baron et al., 2023). Our intervention adopts a combined SH and SE approach to target both knowledge acquisition and actual behavioural change.

Previous interventions often lack detailed procedural reporting, relying on standardized protocols like uniform SE (e.g., going to bed 60 min earlier) or generic SH booklets (Baron et al., 2021; Okul & Fertelli, 2025; Reynolds & Alfano, 2024). The standard intervention limitation is especially relevant for young adults, who experience a biological peak in circadian delay around age 20 (Roenneberg et al., 2004). Generic SH advice often fails to account for this natural drive towards lateness, as well as the diverse behavioural factors (e.g., irregular schedules, technology use) and environmental constraints that further complicate sleep timing (Höhn et al., 2024; Paterson et al., 2019; Xiong et al., 2020). Such factors culminate in bedtime procrastination, where students delay sleep to seek emotion regulation (ER), daily rewards, or social connection (Jeoung et al., 2023). As these motivations are highly individual, effective interventions must move beyond general rules to address the root causes of sleep loss. An effective framework for promoting individualized behavioural change is motivational interviewing (MI), a collaborative, goal‐oriented counselling approach designed to enhance intrinsic motivation and commitment to change (Miller & Rollnick, 2012). MI has been successfully applied to modify sleep patterns by eliciting change talk, exploring ambivalence, and jointly developing actionable plans (Vrabec et al., 2025). Also, MI‐based protocols have proven effective in reducing bedtime procrastination by identifying the psychological drivers of sleep delay and collaboratively developing adaptive alternatives (Jeoung et al., 2023; Suh et al., 2022). Therefore, MI is integrated into our intervention to personalize intervention goals and address individual barriers, thereby enhancing adherence and sustainability.

Sleep loss intensifies amygdala reactivity to negative stimuli and weakens top‐down regulation from the medial prefrontal cortex, leading to heightened negative affect (NA) and impaired ER (Killgore, 2013; Tempesta et al., 2018; Yoo et al., 2007). Evidence from nap studies has shown that rapid eye movement (REM) sleep can modulate emotional perception (Lau et al., 2020) and that sleep gain can enhance inhibitory control over emotional information (Lau et al., 2021). Experimental sleep deprivation consistently reduces positive affect (PA), increases NA, and disrupts ER strategies (Fairholme & Manber, 2015; Tomaso et al., 2021). While the detrimental effects of sleep loss on emotion are well documented, can proactively extending sleep serve as an effective intervention to enhance emotional functions? Understanding the causal efficacy of SE is a necessary first step towards developing accessible, non‐pharmacological strategies for improving emotional health.

The mixed results from observational studies on the sleep–emotion relationship are likely due to differences in measurement tools. While daily diary studies frequently link sleep duration to next‐day affective well‐being (WB), PA, and NA, actigraphy‐based findings remain inconsistent (Hickman et al., 2024). Furthermore, subjective sleep quality often emerges as a more robust predictor of affective experiences and adaptive ER than objective duration alone (Hickman et al., 2024; Parsons et al., 2022). To address the inconsistencies in measurement and sleep metrics, our study examines the effects of both objective (actigraphy‐based) and subjective (diary‐based) sleep measures on emotional outcomes.

While SE is increasingly studied, most research prioritizes physical outcomes like cardiometabolic health (Duraccio et al., 2024) over emotional ones. Findings on emotional impact remain mixed: a one‐week SE pilot improved only PA in young women (Casement et al., 2021), whereas it improved PA and reduced NA in chronic short sleepers (Parsons & Young, 2022). Similarly, five nights of SE enhanced adaptive ER in adolescents (Reynolds & Alfano, 2024), yet no ER changes were observed in chronic short sleepers following intervention (Parsons & Young, 2022). Consequently, further intervention studies are essential to clarify the multicomponent emotional effects of sleep gain.

Building on previous research, this pilot study tests a personalized sleep intervention for chronically short‐sleeping young adults to address two key gaps: the lack of tailored approaches in SE programs and limited evidence on emotional benefits. The protocol consists of a three‐day baseline assessment, a four‐day SH phase providing personalized education and barrier management, and a seven‐day combined phase that maintains hygiene practices and incorporates a 90‐min SE adapted to individual schedules. The compressed three‐four‐seven schedule was adopted for this pilot phase to efficiently evaluate the initial feasibility, intervention flow, and participant engagement before implementing a more extended protocol in the larger‐scale study. Our objectives are as follows:

Objective 1: to examine the intervention's effects on sleep and explore how baseline characteristics (e.g., sleep patterns, psychological traits) moderate the intervention effect on sleep duration.
Objective 2: to investigate the intervention's impact on emotional outcomes, including PA, NA, WB, and ER strategies.
Objective 3: to explore whether improvements in emotional outcomes are predicted by the amount of achieved sleep gain.

## METHODS

### Participants

Participants were recruited from the university community via mass email and the social media platform Rednote. A total of 289 individuals were initially registered for the intervention, and 11 participants (91% female; Mean age ± SD = 25.64 ± 1.5 years) completed the study. Please refer to Figure 1 for details of the exclusion process. Participants provided written informed consent and were enrolled in the intervention. All enrolled participants received the entire intervention.

**FIGURE 1:**
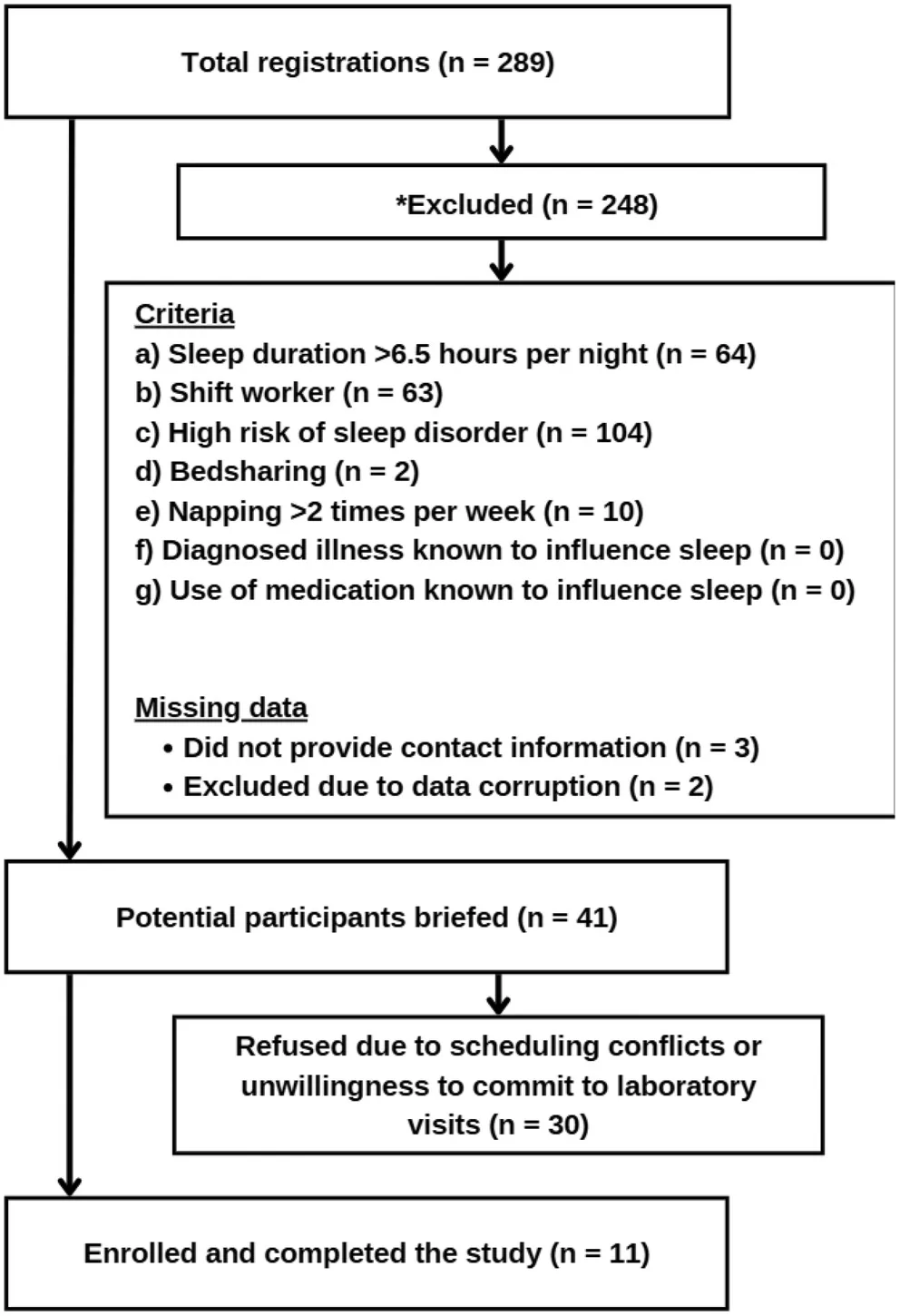
Screening process. (*) The “first‐applicable” exclusion rule is applied in the display of reasons for exclusion. While items f) and g) are shown as 0, this does not represent an absence of diagnosed illnesses or medications in the screened pool. In fact, 25 individuals reported relevant psychiatric (*n* = 11) or physical (*n* = 5) illnesses, or sleep‐altering medications (*n* = 9). However, 24 of these 25 individuals were already excluded by the preceding “high risk of sleep disorder” criteria c, and 1 was excluded by the “sleep duration” criteria a).

The study was approved by the Institutional Review Board of the first author's institution. (#2022–2023‐0141).

Eligibility: (a) average sleep duration <6.5 h past one month; (b) non‐shift worker; (c) no sleep disorders as indicated by the screening interview and Sleep Condition Indicator (Wong et al., 2017); (d) no bedsharing; (e) no more than two naps per week, each lasting less than 90 min; (f) no significant diagnosed physical or mental illness as indicated by self‐reported clinical diagnoses, including psychiatric disorders (e.g., depression, anxiety), chronic conditions (e.g., cardiovascular disease) or major traumatic brain injury and (g) no use of any medication known to influence sleep or affective functions (e.g., melatonin supplements). The rigorous criteria were put in place to ascertain the efficacy of the intervention in nonclinical, generally healthy individuals who are chronic short sleepers.

### Procedure

The intervention procedure lasted 14 days and was divided into three sequential phases: a three‐day baseline phase, a four‐day SH phase and a seven‐day combined phase, during which SE was added to the ongoing SH practices (Figure 2).

**FIGURE 2:**
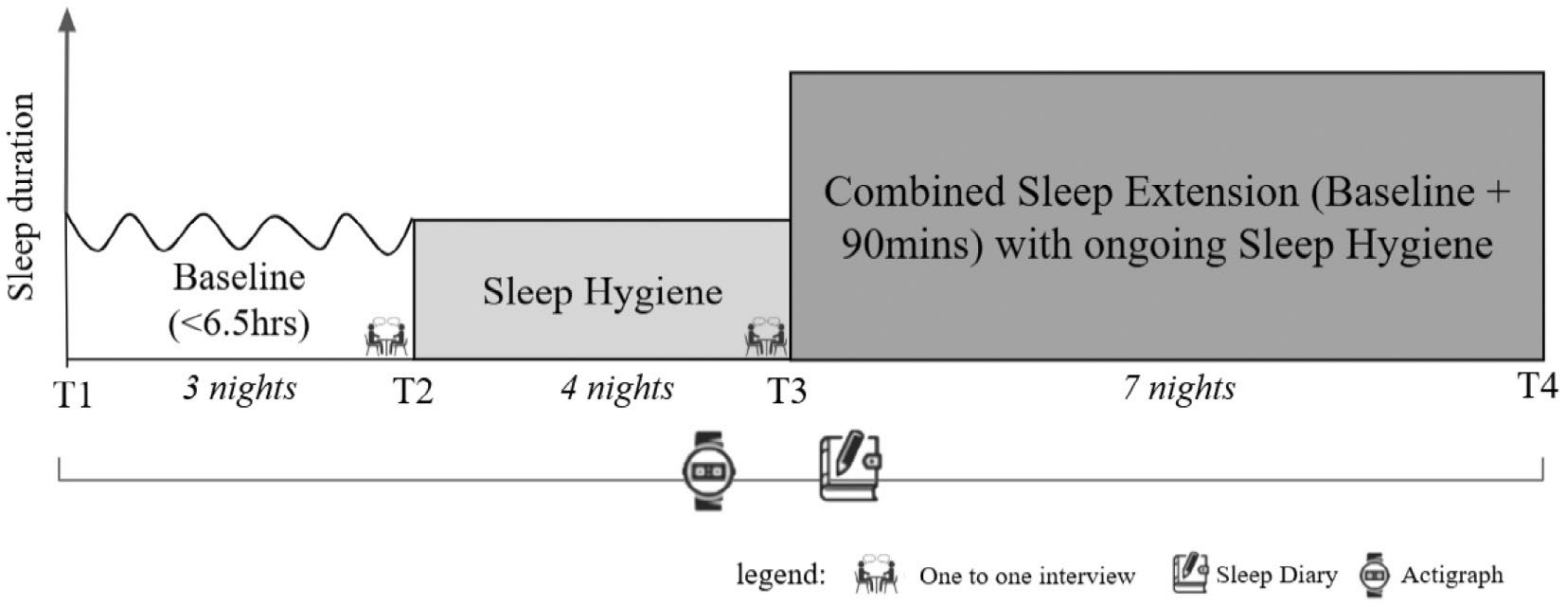
Intervention protocol.

At Time 1 (T1; Baseline): after providing informed consent, participants were instructed to wear an actigraphy watch and complete morning and evening daily diaries throughout the entire intervention period. They also completed the baseline survey on demographic, sleep, and psychological characteristics. The participants maintained their habitual short sleep–wake patterns during the three‐day baseline phase (T1–T2).

At T2, the first interview was conducted, during which a personalized SH plan was collaboratively developed. The participants implemented their individualized SH practices throughout the subsequent SH phase (T2–T3).

At T3, the second interview was conducted. A personalized sleep schedule was collaboratively established with the goal of increasing sleep opportunity by 90 min. The participants followed the personal sleep schedule, in addition to continuing the previously established SH practices throughout the combined phase (T3–T4).

At T4 (post‐test), the participants returned the actigraphy and completed the same survey performed at T1, with postintervention feedback items. The participants were compensated based on their compliance with the intervention protocol, that is, completion of the daily diary and ecological momentary assessments (EMAs). Compliance was encouraged through (a) reminders at T2 and T3 and (b) a proportional compensation system, starting with 10% (HK$30) of the total payment after the baseline, an additional 20% (HK$60) for the SH phase, and the remaining 70% (HK$210) provided only when the protocol was completed.

### Materials

A multimethod and multiple‐timepoint assessment strategy was adopted to comprehensively measure participants' sleep behaviour, affective experiences, and individual differences. Data were collected across three primary sources: (1) survey data: pre‐ and postintervention self‐report questionnaires; (2) daily assessments: objective sleep data, subjective daily diaries and EMAs, and (3) semi‐structured interviews: qualitative data were collected to identify barriers to sufficient sleep, enhance motivation, and develop personalized sleep strategies.

#### Survey

We collected demographic information and variables related to personality, sleep, and psychological characteristics at multiple time points (Table 1). Baseline sleep and personality traits were explored as potential moderators that influence the intervention effect on sleep duration. After the intervention, feedback regarding (a) the overall intervention experience, (b) aspects of SH, (c) experience of SE, (d) satisfaction with the intervention equipment and personnel was collected, and one open‐ended question was given for any additional suggestions or feelings about the intervention (refer to Table S1).

**TABLE 1:** Questionnaire measurements and corresponding time points.

Category	Questionnaire	No. of items	Components/item	Scoring	Measured at
Sleep traits	Chinese Pittsburgh Sleep Quality Index (Tsai et al., 2005)	6	Sleep duration: component 3 (original sleep duration) Sleep quality^a^: the sum of component 1 (subjective sleep quality), component 2 (sleep latency), component 4 (habitual sleep efficiency), component 5 (sleep disturbances), and component 7 (daytime dysfunction)	Hours and minutes; 0–3	T1; T4
	Chronic Sleep Reduction Questionnaire (Dewald et al., 2012)	20	Shortness of sleep; irritation	1–3	T1; T4
	Bedtime Procrastination Scale (Kroese et al., 2014)	9	Sleep habits; bedtime procrastination	1–5	T1; T4
	Subjective Sleep Need	1	How much sleep do you think you need?	Hours and minutes	T1; T4
	Perceived Barriers to Sufficient Sleep^b^ (Paterson et al., 2019)	10	Nine common types of perceived barriers and one open‐ended question of personal barriers	1–5	T2; T3
	Motivation and Attitudes Towards Changing Sleep (Hessler et al., 2018)	9	Motivation for improving sleep	1–5	T2; T3; T4
	Integrated Scale for Sleep Hygiene Practices (Lin et al., 2007; Mastin et al., 2006)	35	Sleep hygiene habits; attitudes; knowledge; tendencies	1–4	T2; T3; T4
	Sleep Self‐efficacy Scale (Johnson, 2018)	9	Confidence in one's own ability to attain good sleep	1–5	T2; T3; T4
Personality traits	International Personality Item Pool (Goldberg, 1992)	50	Extraversion; conscientiousness; agreeableness; intelligence; emotional stability	1–5	T1
	Brief Self‐control Scale (Malouf et al., 2014)	13	General self‐control	1–5	T1
Emotional traits	Depression Anxiety Stress Scale (Lovibond & Lovibond, 1995)	21	Depression; anxiety; stress	0–3	T1; T4
	Positive and Negative Affect Schedule (Watson et al., 1988)	20	Positive affect; Negative affect	1–5	T1; T4
	Perth Emotional Reactivity Scale—Short Form (adapted) (Becerra & Campitelli, 2013)	18	General positive reactivity; General negative reactivity; activation; intensity; duration	1–5	T1; T4
	Emotion Regulation Questionnaire (John & Gross, 2004)	10	Cognitive reappraisal; expressive suppression	1–7	T1; T4

Abbreviation: T, time point.

^a^ The ‘use of sleep medication’ component was excluded as individuals who used any sleep medication were excluded from the study.

^b^ The most prominent barriers to sufficient sleep were combined into an integrated questionnaire scale. The questionnaires at T1 generally showed acceptable to good reliability (Cronbach's *α* = .493 ~ .903).

#### Objective sleep data

Objective sleep–wake patterns were continuously monitored throughout the intervention via a wrist‐worn PRO‐Diary actigraphy (CamNtech). The following key parameters were derived from the raw actigraphy data via MotionWare software and consolidated with subjective sleep diary entries where applicable (Martin & Hakim, 2011):

- Time in bed (TIB): the total duration from lights‐out/attempt to sleep to final awakening and getting out of bed.
- Total sleep time (TST): the total time scored as sleep based on epoch‐by‐epoch analysis.
- Sleep efficiency: the percentage of time asleep while in bed (TST/TIB × 100%).
- Sleep latency (SL): the duration from lights‐out/attempts to sleep to the onset of persistent sleep.
- Fragmentation index: a measure of restlessness during the sleep period, indicating the degree of sleep fragmentation.
- Sleep midpoint: the clock time halfway between sleep onset and the final wake time.

#### Subjective daily assessment

Throughout the entire protocol, the participants completed daily assessments (Table 2). Each morning, they reported on their previous night's sleep via the Consensus Sleep Diary (Carney et al., 2012) on a personal Qualtrics link. Each evening before bed, they reported their daytime affective experiences on the same platform, measuring PA, NA, and overall WB. During the day, they reported their use of adaptive and maladaptive ER strategies in response to random prompts on PRO‐Diary actigraphy, which has inbuilt short questionnaires.

**TABLE 2:** Daily assessments across the study protocol.

Questionnaire	Components/items measured	Scoring	Measured every day at
Consensus Sleep Diary, Items 1–8 (Carney et al., 2012)	Sleep quality (‘How would you rate the quality of your sleep?’) Morning vitality (‘How rested or refreshed did you feel when you woke up for the day?’) Additional items on sleep timing, latency, and disturbances.	Sleep quality: 1 (very poor) to 5 (very good) Morning vitality: 1 (not at all) to 5 (very well‐rested)	After wake
Positive and Negative Affective States (adapted) (Peeters et al., 2003)	Positive affect: happy, enthusiastic, content, relaxed, attentive. Negative affect: upset, annoyed, bored, sad, worried, forgetful.	1 (very slightly or not at all) to 5 (extremely)	Before sleep
World Health Organization—Five Wellbeing Index (Topp et al., 2015)	Five items assessing subjective well‐being over the day (e.g., ‘I have felt cheerful and in good spirits’).	1–5 (never to all the time)	Before sleep
Use of Emotion Regulation Strategies (adapted) (Boemo et al., 2022)	From last beep till now, I… Adaptive strategies: acceptance, distraction, Reappraisal Maladaptive strategies: avoidance, suppression, rumination.	Conditional 6‐point scale: 0 (not relevant/no negative feelings) 1 (strongly disagree) to 5 (strongly agree)	Daytime: 5 random prompts daily (participants required to respond to ≥3), between 9:00 and 21:00.

#### Interview materials on sleep hygiene and sleep extension

We conducted two semi‐structured MI‐based interviews at T2 and T3 (please refer to Table S2 (a) and (b) for the interview questions and procedures). Both sessions were designed to be collaborative and personalized to support behaviour change. The interviews were conducted by one of two interviewers, depending on the participant's mother tongue (Mandarin or Cantonese).

The first interview focused on enhancing participants' awareness of their baseline sleep patterns, identifying barriers to sufficient sleep, and collaboratively developing a personalized SH plan. The interviews followed a structured yet flexible format guided by MI strategies. The key steps were as follows:

- Engagement and review: baseline sleep data from actigraphy and self‐report diaries were reviewed to promote self‐awareness and engagement;
- Behavioural focus and goal setting: participants identified sleep‐related behaviours (e.g., irregular sleep time) they wished to change and proposed the goal (e.g., regular sleep at 01:00 am). Rather than enforcing a standardized early bedtime, we accounted for each participant's chronotype and circadian clock by synthesizing their baseline actigraphy patterns with subjective reports of peak energy levels, preferred wake‐up times on free days, and natural physical fatigue signals. We collaborated with each participant to set sleep–wake goals that matched their circadian clocks and fit their actual daily routines. For example, for ‘night owls’ with late‐night productivity, goals focused on stabilizing a later but consistent sleep–wake schedule (e.g., 03:30–10:30) rather than forcing an unnaturally early bedtime. Conversely, for those with early‐morning inclinations who suffered from evening bedtime procrastination, the goal focused on advancing their bedtime to better align with their natural biological sleepiness and reducing the gap between feeling tired and actually going to sleep.
- Addressing barriers: reasons for sleep restriction were explored, and personal barriers were examined via reflective listening and summarization to facilitate change talk;
- Personalized planning: after viewing a two‐minute SH education video, we cocreated a personalized SH plan, which aimed to stabilize sleep (i.e., reduce sleep variability) and integrate actionable changes into daily routines (e.g., media use curfew 30 min before bed) from T2 to T3.

The second interview focused on building upon prior progress and introducing SE. The key elements included:

- Progress review: we revisited the SH plan set last time, discussing successes and difficulties in implementation;
- Education and reflection: participants watched a psychoeducational video addressing chronic short sleep (e.g., physical and psychological impact) and reflected on its relevance to their own experiences;
- Collaborative planning: we established a tailored 90‐min SE schedule to be executed from T3 to T4 by advancing bedtime, delaying rise time, or a combination of both on the basis of participants' preferences and availability (e.g., later rise times not feasible for participants with morning lessons). The SE plan was integrated with the previously established SH practices.

Across the two interviews, we emphasized participant autonomy and tailored strategies to be relevant within their unique, naturalistic contexts. For example, we negotiated practical rules such as ‘sitting up for non‐sleep activities’ for participants with limited living space. MI strategies such as affirmations, reflections, and goal negotiations helped strengthen commitment and feasibility.

### Analysis plan

Feasibility was assessed through recruitment time, retention rate (the percentage of participants who completed the study), and data completeness. Acceptability was evaluated via the postintervention feedback questionnaire. The daily assessment had a hierarchical structure (729 EMA observations nested within 177 days, nested within 11 participants). The total 177 days exceeded the planned 154 days because some phases were extended due to scheduling conflicts (e.g. limited laboratory availability and participants' schedules, see Table S3 for participant‐specific stage days). To ensure these variations did not bias the results, observations were weighted in the multilevel model to maintain the intended 3:4:7 ratio for the baseline, SH, and SE phases. We used multiple imputation to handle morning and evening missing data (Heymans & Eekhout, 2019; Van Buuren & Groothuis‐Oudshoorn, 2011). EMA prompts with missing items were excluded via listwise deletion (Parsons et al., 2022). The differences in sleep duration between weekdays and weekends were negligible (−0.12 h, *p* = .591). Therefore, we analysed all the data together. The interview data were analysed via reflexive thematic analysis to explore participants' experiences (Braun & Clarke, 2019), with a focus on barriers to sufficient sleep and the strategies used to address them.

#### Quantitative data analysis

To examine the overall effect of the intervention (objective 1), we first conducted *t*‐tests to assess pre–post SE changes, using Cohen's d to represent the standardized difference between time points. Repeated measures ANOVAs were used to test for overall differences in daily sleep outcomes across the three phases (baseline, SH, combined), with Partial *η* ^2^ reported to quantify the proportion of variance accounted for by the intervention stages.

We then fitted eight independent sequential multilevel models (one for each daily sleep outcome) to isolate the effects of each intervention component. We prioritized individual modelling over a multivariate approach because our outcomes involve different units (e.g., minutes for TST vs. Likert‐scale ratings for sleep quality). Independent models allow for a clearer analysis of our primary outcome (TST) separately from other sleep parameters. Each outcome was modelled in three steps:

$$\text{Step}\;1\;\left(\text{Time Trend}\right):{\text{Sleep outcome}}_{\mathrm{ij}}=\beta_{0}+\beta ₁\left({\mathrm{Day}}_{\mathrm{ij}}\right)+u_{0j}+\varepsilon_{\mathrm{ij}}.$$

$$\text{Step}\;2\;\left(\mathrm{SH}\;\text{Effect}\right):{\text{Sleep outcome}}_{\mathrm{ij}}\sim \beta_{0}+\beta ₁\left({\mathrm{Day}}_{\mathrm{ij}}\right)+\beta_{2}\left({\mathrm{SH}}_{j}\right)+u_{0j}+\varepsilon_{\mathrm{ij}}.$$

$$\text{Step}\;3\;\left(\mathrm{SE}\;\text{Effect}\right):{\text{Sleep outcome}}_{\mathrm{ij}}\sim \beta_{0}+\beta ₁\left({\mathrm{Day}}_{\mathrm{ij}}\right)+\beta_{2}\left({\mathrm{SH}}_{j}\right)+\beta_{3}\left({\mathrm{SE}}_{j}\right)+u_{0j}+\varepsilon_{\mathrm{ij}}.$$

*i*: measurement day within participant. *j*: participant ID (*j* = 1…,11). SH_*j*_: a dummy‐coded variable for SH intervention (1 = days from T2–T4, 0 = T1–T2). SE_*j*_: a dummy‐coded variable for SE intervention (1 = days from T3–T4, 0 = T1–T3). *β*_0_: grand intercept, representing the overall mean of the outcome. *β*₁: the fixed effect of time, representing the average daily change in the outcome across the intervention period. *β*_2_: the fixed effect of the SH, representing the average difference in the outcome between the period when SH practices were implemented (T2–T4) and the baseline phase (T1–T2). *β*_3_: the fixed effect of the SE, representing the extra effect of adding the SE protocol on top of the established SH practices. *u*_0*j*_: random intercept for participant *j*. *ε*_ij_: residual error for participant *j* on day *i*.

To explore how baseline characteristics moderated the effect of SE on TST, we extended Step 3 for 13 distinct moderators: TST_ij_ ~ *β*_0_ + *β*₁(Day_ij_) + *β*_2_(SH_*j*_) + *β*_3_(SE_*j*_) + *β*₄(Moderator_*j*_) + *β*₅(SE_*j*_ * Moderator_*j*_) + *u*_0*j*_ + *ε*_ij_. These 13 moderators include baseline sleep duration, baseline sleep quality, chronic sleep reduction, bedtime procrastination, motivation and attitude to change, sleep hygiene practice, sleep self‐efficacy, barrier improvement, sleep debt, conscientiousness, emotional stability, self‐regulation and age.

To examine the intervention's impact on emotional functioning (Objective 2), we applied sequential multilevel models to each of the nine daily emotional outcomes (three affective experiences and six ER strategies), testing the incremental effects of time, SH, and SE. Two multivariate models (one for affective experiences and one for regulation strategies) were conducted to examine the shared and unique effects of the intervention across emotional outcomes.

To explore how sleep gain predicts emotional outcomes (Objective 3), we tested nine multilevel models (one for each emotional outcome) using data from the intervention phases (T2–T4). We first examined the unique contributions of sleep duration and quality:

$$\text{Emotional}\;{\text{Outcome}}_{\mathrm{ij}}\sim \beta_{0}+\beta_{1}\;\left(\text{Between Sleep}\;{\text{Gain}}_{j}\right)+\beta_{2}\left(\text{Within Sleep}\;{\text{Gain}}_{ij}\right)+\beta_{3}\left(\text{Sleep}\;{\text{Quality}}_{j}\right)+u_{\mathrm{oj}}\;+\varepsilon_{\mathrm{ij}}$$

Between Sleep Gain_*j*_: participant *j*'s average sleep duration during the intervention phases minus their baseline average. Within Sleep Gain_ij_: the deviation of participant *j*'s sleep duration on day *i* from their own average during the intervention phases. Sleep Quality_*j*_: average daily sleep quality rating across the intervention phases.

The model tested whether individuals who sleep more on average feel better (*β*₁), whether a person feels better on days after they obtain more sleep than their own average (*β*_2_), and whether these associations are independent of sleep quality (*β*_3_). The false discovery rate (FDR) across the multiple models for each research objective was controlled at .05 via a two‐stage linear step‐up procedure (Benjamini et al., 2006). This procedure was independently applied to: the eight sleep outcomes (Step 3) and the 13 moderator tests for TST (Objective 1), the nine emotional outcomes regarding the SE intervention effect (Step 3, Objective 2), and the nine emotional outcomes regarding within‐person sleep gain effects (Objective 3).

We further conducted exploratory mediation analyses with daily level data (Falk et al., 2024) to examine whether the effect of sleep duration on emotions operated through improvements in sleep quality (18 model sets in total, two per emotional outcome). We tested (a) the absolute level model, that is, whether daily sleep quality mediated the relationship between daily sleep duration and next‐day emotions, and (b) the gain score model, that is, whether nightly gains in sleep quality (relative to baseline) mediated the relationship between nightly sleep duration gains and emotions.

All multilevel models accounted for individual differences. For the ER strategies (assessed multiple times daily) models, the random intercepts for both the participant and the day were added to account for between‐person and between‐day differences (Parsons et al., 2022). In addition, multilevel models were applied to all imputed datasets, with the results pooled via Rubin's rules. Continuous predictors were grand‐mean (person‐level) or group‐mean (daily level) centered. Cohen's *f* ^2^ was used to quantify the level of the intervention effect (Schiller et al., 2017), with thresholds of small (0.02), medium (0.15), and large (0.35) (Selya et al., 2012). We reported conventional effect sizes for each test (Cohen's *d* for *t*‐test, Partial *η* ^2^ for ANOVA, Cohen's *f* ^2^ for multilevel) to support direct comparison with earlier work in the field.

To address statistical power considerations, post hoc G*Power analyses suggested that our sample of 11 participants provided 77% power to detect the observed pre‐ and postintervention changes in sleep outcomes (Figure S1). For multilevel models, we conducted simulation‐based power analyses for SE effects on primary outcomes via the ‘simr’ package (Green & MacLeod, 2016). Models were extended along the participant dimension to examine statistical power across sample sizes ranging from 10 to 60 participants. Power analyses focused on the main intervention effects to inform sample size planning for future efficacy trials. Moderation and mediation analyses are presented as exploratory, as detecting such complex effects typically requires larger samples, approximately 40–50 participants on the basis of sample sizes in comparable intensive longitudinal sleep studies examining within‐person mechanisms (Junça‐Silva et al., 2025).

Monte Carlo simulations indicated that our pilot sample of 11 participants provided 36–87% power to detect SE effects across different outcomes, whereas sample sizes of 20–30 participants would achieve adequate power (≥80%) for most primary outcomes (Figure S2). All models were examined in R, mainly with the ‘lmer’, ‘mice’, ‘multilevelmediation’, and ‘simr’ packages.

#### Interview data

The reflexive thematic analysis focused on three key aspects: barriers to sufficient sleep, strategies utilized to address insufficient sleep, and the perceived usefulness of these strategies. The qualitative notes were first translated into English and formatted. The translated notes were assigned labels on the basis of the identified theme of the content. The identified themes were then reviewed, refined, and combined to reduce conceptual overlap. Two researchers independently reviewed the notes and conducted the coding process. The interrater reliability coefficient (Cohen's kappa = 1) indicated complete agreement. Code saturation was measured with the Code Frequency Counts approach (Hennink & Kaiser, 2022), whereby the cumulative number of unique thematic categories was tracked across successive interviews. Starting from the first participant, the cumulative number of unique categories (i.e., barriers) was tracked across successive interviews.

## RESULTS

### Feasibility, acceptance, and preliminary effects of the sleep intervention

The targeted sample of 11 participants was recruited within 40 days. All participants successfully completed the entire intervention within 54 days. The participants showed high adherence to the intervention procedures, as evidenced by the completion of daily sleep diaries and actigraphy monitoring. Specifically, 1% of morning diaries and 8.26% of evening diaries were missing, with EMA missing data ranging from 0.28 to 2.47%. Regarding acceptability, participants expressed strong overall satisfaction (M = 4.55/5), with all participants reporting a willingness to maintain extended sleep habits postintervention. All participants acquired sleep knowledge, and 91% perceived sleep improvement. Although 55% of the participants reported that it was challenging to extend their sleep duration, all of them felt comfortable with extended sleep and reported perceived benefits in both cognitive function and mood. The success in developing personalized strategies was captured by one participant's feedback: ‘I found a method that works for me to increase my sleep time’ (refer to Table S1 for detailed feedback results).

The overall and individual changes in sleep duration across the intervention phases are shown in Figures S3 and S4. The pre‐ and postsurvey assessments showed that after the intervention, the participants reported increased sleep duration (mean difference = 0.75 h, 95% CI [0.25, 1.25], Cohen's *d* = 1.08), less bedtime procrastination (mean difference = −6.28, 95% CI [−11.59, −2.05], Cohen's *d* = −.96) and fewer perceived barriers to sufficient sleep (mean difference = −4.10, 95% CI [−7.02, −1.06], Cohen's *d* = −.91). Furthermore, participants followed more SH practices after the SH period (mean difference = 15.55, 95% CI [2.29, 28.81], Cohen's *d* = 0.94) (Tables 3 and 4). The three‐phase comparison results showed that TIB (*F* = 52.90, *p* < .001) and TST (*F* = 30.14, *p* < .001) significantly increased from baseline to subsequent phases and that sleep quality improved across phases (*F* = 5.12, *p* = .007). However, no significant phase‐level differences were observed for sleep efficiency (*p* = .883), sleep latency (*p* = .853), fragmentation index (*p* = .343), and sleep midpoint (*p* = .129; see Table 5).

**TABLE 3:** Paired *t*‐tests of sleep variables from baseline (T2) to post‐sleep extension (T4).

Sleep variable	Mean (SD)		Paired *t*‐test		95% CI	
	Pre	Post	*t* (df)	*p*	Lower	Upper
Sleep quality	5.36 (1.75)	5.00 (1.34)	.77 (10)	.459	−0.69	1.42
Sleep duration	6.28 (0.49)	7.03 (0.85)	−3.38 (10)	.007**	−1.25	−0.25
Chronic sleep reduction	38.03 (6.03)	33.91 (6.61)	1.89 (10)	.088^†^	−0.73	8.91
Bedtime procrastination	38.18 (4.71)	31.36 (7.67)	3.19 (10)	.010*	2.05	11.59
Subjective sleep need	7.80 (0.56)	8.50 (0.67)	−2.05 (9)	.07^†^	−1.53	0.08
Perceived barriers (T2 × T3)	26.10 (2.95)	22.00 (5.42)	3.01 (10)	.013*	1.06	7.12

*Note*: ^†^ *p* < .10, *p < .05, **p < .01.

**TABLE 4:** ANOVA and post‐hoc Tukey HSD tests for behaviour change variables across T2–T4.

Variable	*F* value	*p*	Partial *η* ^2^	Post‐hoc comparisons (Tukey HSD)	95% CI	
					Lower	Upper
Motivation and attitudes towards changing sleep	*F* (2, 20) = 0.651	0.532	0.061	T2 versus T3: −2.00, *p* = .761	−9.13	5.13
				T2 versus T4: −3.18, *p* = .508	−10.31	3.95
				T3 versus T4: −1.18, *p* = .908	−8.31	5.95
Sleep hygiene practices	*F* (2, 20) = 4.712	0.021*	0.32	T2 versus T3: 15.55, *p* = .020*	2.29	28.81
				T2 versus T4: 11.36, *p* = .102	−1.90	24.62
				T3 versus T4: −4.18, *p* = .709	−17.44	9.08
Sleep self‐efficacy	*F* (2, 20) = 0.203	0.818	0.02	T2 versus T3: −1.18, *p* = .946	−10.6	8.20
				T2 versus T4: −2.36, *p* = .802	−11.7	7.02
				T3 versus T4: −1.18, *p* = .946	−10.6	8.20

* *p* < .05.

**TABLE 5:** Sleep change across three phases.

Sleep variables	Baseline phase mean (SD)	SH phase mean (SD)	Combined phase mean (SD)	*F*	*p*
Time in bed	6.22 (1.13)	7.04 (1.26)	8.38 (1.17)	52.903	<.001***
Total sleep time	5.01 (1.13)	5.62 (1.26)	6.67 (1.21)	30.144	<.001***
Sleep efficiency	80.60 (10.27)	79.80 (11.47)	79.63 (10.58)	0.1242	.883
Sleep latency	13.06 (20.18)	11.96 (21.38)	10.95 (20.17)	0.1594	.853
Fragmentation index	27.86 (14.75)	29.93 (15.87)	32.24 (17.71)	1.0779	.343
Sleep midpoint	05:32 (01:23)	05:03 (01:22)	05:06 (01:13)	2.0733	.129
Sleep quality	3.19 (0.70)	3.23 (0.90)	3.62 (0.87)	5.1151	.007**
Morning vitality	3.02 (1.00)	3.15 (0.92)	3.14 (0.89)	2.716	.069^†^

*Note*: ^†^ *p* < .10, **p < .01, ***p < .001.

### Objective 1: Sleep hygiene and sleep extension effects on sleep

Descriptions of the variance of multilevel variables are found in Table S4. The time‐only model (Step 1) showed that TIB and TST increased steadily across days, accompanied by improvements in subjective sleep quality and morning vitality. No day‐to‐day changes were observed for the fragmentation index, SL, sleep midpoint, or sleep efficiency. After adding the SH (Step 2), there was greater morning vitality and a trend towards increased sleep fragmentation. The inclusion of SE (Step 3) provided additional improvements on top of SH. Specifically, while SH significantly increased TIB (+0.72 h, *p* = .019, Cohen's *f* ^2^ = 0.06), SE further extended TIB (+1.49 h, *p* < .001, Cohen's *f* ^2^ = 0.09) and tended to increase TST (*β* = 0.55 h, *p* = .059). Morning vitality was consistently enhanced by both SH (*β* = 0.52, *p* < .001, Cohen's *f* ^2^ = 0.03) and SE (*β* = 0.32, *p* < .001, Cohen's *f* ^2^ = 0.08). However, both SH and SE were associated with lower sleep efficiency and more fragmentation, suggesting that longer sleep duration comes at the cost of deteriorated sleep continuity. In contrast, no significant intervention effects of either SH or SE were observed for subjective sleep quality, sleep latency, or sleep midpoint (Table 6). Overall, SH primarily improved subjective morning vitality, whereas SE produced the strongest effects by substantially extending sleep duration and further enhancing morning vitality, but at the cost of reduced sleep efficiency and continuity. The baseline moderator exploratory results were in Table S5.

**TABLE 6:** Sleep hygiene and sleep extension intervention effect on sleep.

Outcome variable	Predictor	Step 1: Time only	Step 2: + SH	Step 3: + SE
Time in bed (h)	Day	0.161 (0.020)***	0.134 (0.028)***	−0.022 (0.036)
	SH		0.403 (0.327)	0.719 (0.303)*
	SE			1.492 (0.299)***
Total sleep time (h)	Day	0.139 (0.017)***	0.141 (0.025)***	0.084 (0.038)*
	SH		−0.044 (0.278)	0.067 (0.282)
	SE			0.550 (0.290)^†^
Subjective sleep quality	Day	0.041 (0.013)**	0.042 (0.019)*	0.021 (0.031)
	SH		−0.020 (0.203)	0.015 (0.209)
	SE			0.212 (0.217)
Morning vitality	Day	0.038 (0.006)***	0.035 (0.008)***	−0.027 (0.015)^†^
	SH		0.435 (0.098)***	0.518 (0.102)***
	SE			0.324 (0.107)**
Sleep efficiency (%)	Day	0.017 (0.146)	0.287 (0.207)	0.846 (0.322)**
	SH		−4.255 (2.311)^†^	−5.290 (2.330)*
	SE			−5.486 (2.403)*
Fragmentation index	Day	0.233 (0.238)	−0.187 (0.335)	−1.142 (0.518)*
	SH		6.653 (3.798)^†^	8.382 (3.829)*
	SE			9.435 (3.855)*
Sleep latency (min)	Day	−0.020 (0.280)	−0.050 (0.378)	−0.007 (0.521)
	SH		0.534 (4.547)	0.535 (4.562)
	SE			−0.553 (4.454)
Sleep midpoint (minutes past midnight)	Day	−1.185 (0.948)	−1.293 (1.346)	−3.519 (2.155)
	SH		1.675 (15.286)	6.225 (15.612)
	SE			21.178 (16.019)

*Note*: The table presents unstandardized coefficients with standard errors in parentheses. *p*‐values for the final intervention effects (Step 3) were adjusted using a two‐stage linear step‐up procedure across the eight outcomes. Coefficients for Time in Bed, Total Sleep Time, Morning Vitality, Sleep Efficiency, and Fragmentation Index remained significant after false discovery rate correction. ^†^ *p* < .10, *p < .05, **p < .01, ***p < .001.

Abbreviations: SH, sleep hygiene; SE, sleep extension.

### Objective 2: Intervention effects on affective experiences and emotion regulation

The trait‐level emotions did not differ pre‐ and post SE intervention (Table S6). In the sequential modelling (Table 7), affective experience and regulation improved progressively over time (Step 1), while most outcomes showed no additional improvement from SH alone (Step 2). However, the distinct contributions of SH and SE became clear in the Step 3 models. Specifically, SH significantly improved WB (*β* = 2.022, *p* = .017, Cohen's *f* ^2^ = 0.02) compared with baseline. Beyond the SH effect, SE provided incremental benefits by further increasing WB (*β* = 2.043, *p* = .024, Cohen's *f* ^2^ = 0.02), enhancing PA (*β* = 1.601, *p* = .032, Cohen's *f* ^2^ = 0.02), decreasing NA (*β* = −1.242, *p* = .060) and improving ER strategies such as acceptance (*β* = 0.380, *p* = .018, Cohen's *f* ^2^ = 0.02) and distraction (*β* = 0.358, *p* = .014, Cohen's *f* ^2^ = 0.00).

**TABLE 7:** Single model analysis of intervention effects on emotional outcomes.

Outcome	Predictor	Step 1: Time only	Step 2: +SH	Step 3: +SE
Well‐being	Day	0.169 (0.054)**	0.068 (0.075)	−0.147 (0.122)
	SH		1.579 (0.826)^†^	2.022 (0.838)*
	SE			2.043 (0.896)*
Positive affect	Day	0.093 (0.043)*	0.065 (0.061)	−0.102 (0.100)
	SH		0.439 (0.685)	0.775 (0.698)
	SE			1.601 (0.740)*
Negative affect	Day	−0.151 (0.040)***	−0.131 (0.058)*	−0.002 (0.091)
	SH		−0.300 (0.626)	−0.560 (0.640)
	SE			−1.242 (0.656)^†^
Emotion regulation—Acceptance	Day	−0.005 (0.009)	−0.006 (0.014)	−0.046 (0.022)*
	SH		0.026 (0.152)	0.113 (0.153)
	SE			0.380 (0.160)*
Emotion regulation—Distraction	Day	0.006 (0.008)	0.019 (0.012)	−0.019 (0.020)
	SH		−0.190 (0.135)	−0.101 (0.138)
	SE			0.358 (0.145)*
Emotion regulation—Avoidance	Day	−0.036 (0.008)***	−0.043 (0.013)***	−0.024 (0.021)
	SH		0.100 (0.141)	0.057 (0.145)
	SE			−0.181 (0.152)
Emotion regulation—Suppression	Day	−0.016 (0.008)*	−0.033 (0.012)**	−0.010 (0.019)
	SH		0.257 (0.127)	0.204 (0.131)
	SE			−0.218 (0.138)
Emotion regulation—Reappraisal	Day	0.004 (0.008)	0.005 (0.013)	0.011 (0.020)
	SH		−0.008 (0.139)	−0.022 (0.144)
	SE			−0.058 (0.151)
Emotion regulation—Rumination	Day	−0.027 (0.008)***	−0.032 (0.012)**	−0.014 (0.019)
	SH		0.082 (0.132)	0.041 (0.135)
	SE			−0.176 (0.142)

*Note*: The table presents unstandardized coefficients with standard errors in parentheses. *p*‐values for the final intervention effects (Step 3) were adjusted using a two‐stage linear step‐up procedure across the nine outcomes. No significant effects in these independent models remained significant after false discovery rate correction. ^†^ *p* < .10, *p < .05, **p < .01, ***p < .001.

Abbreviations: SH, sleep hygiene; SE, sleep extension.

The multivariate model (Table 8) confirmed that SE significantly improved WB (*β* = 2.430, *p* = .007) and PA (*β* = 2.253, *p* = .012) relative to NA, whereas no significant effect was found for NA (*p* = .25). Regarding ER, the multivariate model showed a significant interaction effect between SE phase and strategy type (*β* = .427, *p* < .001), suggesting that the effect of SE differed for maladaptive strategies compared with adaptive strategies. Decomposition of the interaction showed that SE not only enhanced adaptive strategy use (*β* = .230, *p* = .003) but also reduced maladaptive strategy engagement (*β* = −.198, *p* = .010).

**TABLE 8:** Multivariate analysis of intervention effects on emotional outcomes.

Parameter	*β* (SE)	*p*
Affective experiences (reference: NA)		
Main effects		
(a) Day	−0.06 (0.08)	.42
(b) SH	−0.29 (0.84)	.727
(c) SE	−0.88 (0.77)	.25
Intervention contrasts		
(a) SH on WB versus NA	1.95 (1.09)	.075^†^
(b) SH on PA versus NA	0.90 (1.08)	.408
(c) SE on WB versus NA	2.43 (0.90)	.007**
(d) SE on PA versus NA	2.25 (0.89)	.012*
Emotional regulation (reference: adaptive strategies)		
Main effects		
(a) Day	−0.017 (0.010)	.078^†^
(b) SH	0.107 (0.077)	.163
(c) SE	0.230 (0.077)	.03*
Intervention contrasts		
(a) SH on maladaptive versus adaptive	−0.125 (0.072)	.083^†^
(b) SE on maladaptive versus adaptive	−0.427 (0.059)	<.001***

*Note*: The table presents unstandardized coefficients with standard errors in parentheses. ^†^ *p* < .10, *p < .05, **p < .01, ***p < .001.

Abbreviations: NA, negative affect; PA, positive affect; SH, sleep hygiene; SE, sleep extension; WB, Wellbeing.

Overall, while the specific SE effects in individual models did not reach the adjusted significance threshold after FDR correction, the multivariate findings suggested that SE may optimize emotional functioning by bolstering positive experiences and simultaneously promoting adaptive strategies while discouraging maladaptive ones.

### Objective 3: Sleep gain effects on emotional outcomes

On days when participants obtained more sleep than intervention average improvement, they showed significant next‐day improvements in positive affective experiences, including higher WB (*β* = 0.578, *p* = .016, Cohen's *f* ^2^ = 0.01) and PA (*β* = 0.511, *p* = .007, Cohen's *f* ^2^ = 0.02), with a trend towards reduced NA (*β* = −0.323, *p* = .081. Cohen's *f* ^2^ = 0.04). The participants who reported higher average sleep quality throughout the intervention period also reported significantly better WB (*β* = 4.271, *p* = .009, Cohen's *f* ^2^ = 0.33) and less NA (*β* = −3.535, *p* = .004, Cohen's *f* ^2^ = 0.37). Within‐person sleep gain predicted increased use of adaptive ER strategies, including acceptance (*β* = 0.087, *p* = .042, Cohen's *f* ^2^ = 0.02) and reappraisal (*β* = 0.094, *p* = .012, Cohen's *f* ^2^ = 0.00), and a reduction in rumination (*β* = −0.152, *p* < .001, Cohen's *f* ^2^ = 0.04, Table 9). In contrast to the robust within‐person effects, between‐person sleep gain was not a significant predictor of any outcome in this sample (all *p* > .05), suggesting that the observed emotional benefits were specifically tied to within‐person fluctuations rather than stable differences in total sleep gain from the intervention.

**TABLE 9:** Sleep gain effect on emotional outcomes.

Outcome variable	Predictor	*β* (SE)	*p*
Well‐being	(intercept)	−1.960 (5.641)	.729
	Between sleep gain	1.207 (1.221)	.325
	Within sleep gain	0.578 (0.236)	.016^†^
	Sleep quality	4.271 (1.603)	.009**
Positive affect	(intercept)	9.453 (5.575)	.093^†^
	Between sleep gain	−0.022 (1.211)	.985
	Within sleep gain	0.511 (0.187)	.007**
	Sleep quality	1.889 (1.588)	.237
Negative affect	(intercept)	21.920 (4.170)	<.001***
	Between sleep gain	0.480 (0.935)	.609
	Within sleep gain	−0.323 (0.183)	.081^†^
	Sleep quality	−3.535 (1.189)	.004**
Acceptance	(intercept)	5.331 (1.339)	<.001***
	Between sleep gain	−0.397 (0.288)	.168
	Within sleep gain	0.087 (0.043)	.042*
	Sleep quality	−0.493 (0.381)	.196
Distraction	(intercept)	0.768 (2.773)	.782
	Between sleep gain	−0.481 (0.597)	.421
	Within sleep gain	0.066 (0.036)	.069^†^
	Sleep quality	0.611 (0.790)	.439
Avoidance	(intercept)	2.698 (3.218)	.402
	Between sleep gain	−0.727 (0.695)	.296
	Within sleep gain	−0.060 (0.037)	.111
	Sleep quality	0.141 (0.916)	.878
Suppression	(intercept)	1.748 (2.500)	.485
	Between sleep gain	−0.683 (0.540)	.207
	Within sleep gain	−0.059 (0.036)	.105
	Sleep quality	0.218 (0.712)	.759
Reappraisal	(intercept)	2.549 (2.362)	.281
	Between sleep gain	0.041 (0.512)	.936
	Within sleep gain	0.094 (0.037)	.012*
	Sleep quality	0.014 (0.673)	.983
Rumination	(intercept)	1.743 (2.256)	.44
	Between sleep gain	−0.515 (0.488)	.294
	Within sleep gain	−0.152 (0.035)	.001***
	Sleep quality	0.127 (0.642)	.843

*Note*: The table presents unstandardized coefficients with standard errors in parentheses. *p*‐values for within‐person sleep gain effects were adjusted using a two‐stage linear step‐up procedure across the nine emotional outcomes. Within‐person effects for Well‐being, Positive Affect, Acceptance, Reappraisal, and Rumination remained significant after false discovery rate correction. ^†^ *p* < .10, *p < .05, **p < .01, ***p < .001.

Exploratory mediation analyses tested whether these emotional benefits operated through improvements in sleep quality (Table S7). In both the absolute level and gain score models, sleep duration consistently predicted better sleep quality (all paths were significant). However, sleep quality did not significantly mediate the relationship between sleep duration and emotional outcomes in any model (all indirect effects were nonsignificant), suggesting that the emotional benefits of extended sleep may operate through mechanisms independent of improvements in perceived sleep quality. The results require verification in future studies with adequate power to detect mediation.

### Qualitative insights: barriers, strategies, and processes of change

The participants reported individual barriers to sufficient sleep. While behavioural factors such as excessive phone use before sleep were nearly universal (91%), the underlying reasons varied widely. Some participants used electronic devices habitually, whereas others used them as emotional comfort or compensation for daytime stress. Pre‐sleep arousal (45%), such as anxiety, depression or rumination in bed, often interfered with sleep onset. Environmental constraints (55%), such as roommate noise or bed‐sharing, were seen as largely immutable. Additionally, nighttime eating (18%) and a high workload (18%) may delay sleep. Most participants expressed a willingness to change their bedtime routines, but changing their sleep environment was quite challenging and was constrained by their objective living conditions.

Successful strategies involved context‐based habit change strategies (e.g., phone distancing or app limits) rather than substitution‐based approaches (e.g., replacing phone use with music or reading) to reduce bedtime phone use, which participants reported difficulty initiating and maintaining. A frequently reported strategy was bedside journaling to minimize the influence of negative pre‐sleep thoughts, allowing the cognitive offloading of intrusive thoughts before sleep. While this method was effective for many, at least one participant resisted journaling, stating, ‘I'm just someone who processes everything internally,’ showing the role of individual cognitive style in moderating the strategy's adoption. In contrast, attempts to alter environmental barriers (e.g., using earplugs, reorganizing sleep spaces) had limited effectiveness, showing the constraints faced by participants living in shared or noisy conditions. Only sitting up and using half the bed for non‐sleep activities worked for one participant, suggesting that a bed–sleep association may be helpful. The detailed information concerning the barriers and strategies is provided in Table 10.

**TABLE 10:** Summary of sleep barriers and participant‐reported coping strategies.

Category	Specific barrier description	Strategy implemented	Efficacy at follow‐up (T3)
Bedtime digital use	Mindless phone scrolling in bed after lying down.	Placing the phone away from bed before sleep.	Effective.
	Pre‐sleep TikTok use causes overstimulation.	Setting an alarm to put their device down.	Effective.
	Compulsive use of short‐form content (TikTok, Rednote).	Self‐imposed deadlines for phone use.	Ineffective. Rarely followed the deadline
	Watching games and TV series in bed.	Placing phone under the bed upon getting into bed.	Partially effective: reduced phone use but introduced a longer sleep latency period.
	Scrolling through social media apps before sleep.	Using app time limits (midnight‐6 am) and removing phone from bedroom.	Effective.
	Compensatory late‐night phone use serves as a way to reclaim personal time lost to a demanding daytime workload.	Replacing phone use with relaxing music 20 min before bed.	Ineffective, to try again with music/ reading 20 min before bed.
	Pre‐sleep phone use for entertainment.	Substituting phone use with reading.	Ineffective. Reported at T3: did more work instead of using the phone.
	Phone use before sleep as habit	Keeping a nightly journal to replace using phone before sleep.	No record.
	1. Mood‐dependent online chatting with strangers. 2. The phone is kept close to the body as it provides a sense of safety. 3. Needs to hold the phone to fall asleep.	Setting an alarm to put their device down.	Ineffective. Attributed to ‘fate’; exhibited low motivation and an external locus of control.
	Pre‐sleep media use	Limiting app usage time.	No record.
Sleep environment	Interruption by roommate.	No viable strategy identified.	Unchanged.
	Chatting with roommate late at night; home renovations.	No viable strategy identified.	Unchanged.
	Cluttered room with many sleep‐unrelated items.	No viable strategy identified (room too small).	Unchanged.
	Noisy neighbours especially on weekends.	Using earplugs.	Strategy in place but ineffective.
	Frequently awakened by roommate's alarm and unable to return to sleep.	No viable strategy identified.	Unchanged.
	Multi‐purpose use of bed in a small living space (e.g., eating, using phone).	Sitting up in bed for all non‐sleep activities (i.e., bed sectioning).	Effective.
Work/study stress	Stress from deadlines and coursework affecting sleep quality.	No viable strategy identified.	Unchanged.
	Workload and deadlines delaying bedtime.	Journaling with next day planning to manage stress and organize time.	Effective. Reported reduced anxiety and improved subjective sleep quality.
Pre‐sleep arousal	Overthinking next‐day plans or past experiences in bed.	Bedside journaling to ‘unload’ thoughts.	Effective.
	Depressed and anxious mood leading to prolonged pre‐sleep thoughts.	No record.	No record.
	Negative mood and rumination before sleep.	Journaling to process emotions and thoughts.	Effective.
	Constant, near‐constant rumination and overthinking.	Attempted journaling.	Ineffective. Prefer to internally process thoughts
	Bad mood/rumination leading to later sleep times.	Drinking longan ginger tea at 11 pm as a relaxing habit.	Reported as a habitual practice.
Other	1. Late‐night social activities (e.g., mahjong). 2. Drinking close to bedtime.	Reported strong self‐control to adhere to sleep hygiene protocols.	Effective.
	Frequent late‐night food cravings and eating leading to delayed sleep.	Drinking longan ginger tea to induce sleepiness and suppress cravings.	Ineffective in fully curbing cravings or preventing delayed sleep.

*Note*: Each row represents a specific barrier for a participant; each participant may have multiple barriers.

## DISCUSSION

The pilot study showed that the intervention was highly feasible and acceptable, with significant improvements in sleep duration, morning vitality, and key emotional outcomes. SH primarily improved subjective vitality, whereas the addition of SE was critical for achieving substantial gains in sleep duration and emotional benefits. Notably, the emotional benefits were mostly linked to a night‐to‐night increase in sleep duration, independent of perceived sleep quality. However, these gains in sleep quantity were accompanied by a daily‐level decline in objective sleep efficiency and an increase in fragmentation.

### Sleep intervention feasibility, efficacy, and trade‐offs

The high retention, compliance, and satisfaction confirmed the feasibility and acceptability of our personalized sleep intervention. The high degree of engagement translated into meaningful behavioural change, specifically, a significant improvement in SH practices following the SH period and significant reductions in bedtime procrastination and perceived barriers to sufficient sleep postintervention. Beginning with a tailored SH and progressing to SE proved effective. While SH alone was associated with a modest increase in TIB, SE was the primary driver of substantial sleep gain, adding nearly 1.5 h of TIB beyond that achieved by hygiene practices alone. The result aligned with reviews showing that combining direct behavioural prescriptions with indirect education is more effective than indirect education alone (Baron et al., 2021; Gadam et al., 2023). The magnitude of SE is notable, exceeding the amount reported in previous studies (an average increase of 45 min) (Baron et al., 2021), and may be attributed to the personalized and motivational nature of our intervention. Rather than providing generic advice, our tailored approach addressed individual barriers through collaboratively setting feasible goals, which likely increased participant buy‐in and adherence (Gadam et al., 2023).

A notable finding was the trade‐off between increased sleep duration and reduced sleep efficiency and increased fragmentation, as detected by the daily level multilevel models. Multilevel findings contrast with trait‐based evidence linking longer sleep to superior continuity in healthy adults (Kishi et al., 2017). This discrepancy likely arises from our study's focus on experimental extension rather than natural sleep patterns; our participants were chronically sleep‐restricted, averaging 6.28 h at baseline despite a 7.80 h subjective need. In such individuals, active sleep extension may disrupt a chronic equilibrium adapted to sleep loss. When the body starts to repay accumulated sleep debt, the sleep system undergoes a recalibration phase where it resets its natural timing and depth before sleep becomes stable again (Åkerstedt et al., 2009). Notably, despite the observed objective fragmentation in daily models, participants reported consistently enhanced morning vitality and improved phase‐level subjective sleep quality. This suggests that in chronically sleep‐restricted individuals, the subjective feeling of restfulness may begin to recover even while objective sleep continuity is undergoing a period of recalibration.

### Intervention effects on emotional functioning and the role of sleep gain

Although individual emotional outcomes did not meet the FDR‐adjusted significance threshold, multivariate results and consistent directional trends suggest that SE bolsters positive experiences while simultaneously enhancing adaptive and reducing maladaptive strategies. This pattern was consistent with prior research showing SE‐induced improvements in morning PA (Casement et al., 2021). The lack of individual significance likely reflects the conservative nature of FDR correction in a small pilot sample rather than a lack of impact, especially as the multivariate interaction confirmed that SE benefits were distributed across both affective states and regulation processes.

Stronger evidence emerged from the daily sleep gain (Objective 3), where within‐person sleep gain remained a significant predictor across five outcomes (WB, PA, Acceptance, Reappraisal, and Rumination) even after FDR correction. This suggests that emotional improvements are driven by nights when individuals exceed their own average sleep duration, an effect independent of perceived sleep quality. While subjective sleep quality is a known, consistent predictor of affect (Duraccio et al., 2024; Parsons et al., 2022) and correlated with WB and NA in our data, extra sleep duration produced incremental benefits beyond perceived quality. This pattern likely reflects the restoration of prefrontal–amygdala functional connectivity, which enhances top‐down emotional control and psychological strength (Killgore, 2013). Such neural pathways appear particularly responsive to increased sleep opportunity in chronically sleep‐restricted individuals.

The observed effect sizes were modest, which is consistent with the SE literature, where emotional benefits are generally incremental compared with the pronounced effects of sleep loss (Parsons & Young, 2022). Effect sizes derived from multilevel models (Cohen's *f* ^2^) provide a more conservative estimate than traditional pre‐post comparisons (Cohen's *d*), as they isolate the unique incremental contribution of SE after accounting for nested variances and time trends. Sleep interventions typically have robust effects on primary sleep outcomes but more variable effects on secondary psychological outcomes (Griggs et al., 2020). Despite their modest scale, the consistency of these gains across multivariate and within‐person analyses suggests that SE produces meaningful, incremental benefits that may become more pronounced and persistent for chronically short sleepers over time.

### Implications for intervention targeting, personalization, and research methodology

The rigorous screening, while limiting generalizability, successfully identified a unique sample of ‘pure’ chronic short sleepers. By focusing on pure short sleepers, we could observe the intervention's feasibility and its relationship with emotional health without the immediate interference of major clinical illnesses. Notably, the screening process suggested a significant degree of comorbidity; almost all participants who reported diagnosed psychiatric or physical conditions were captured by the ‘high risk of sleep disorder’ criteria. The proportion of health conditions was consistent with epidemiological findings regarding mental health prevalence among young Chinese adults (Huang et al., 2019), suggesting that sleep disturbances are often a prominent feature of underlying distress. The high exclusion rate due to sleep disorder risk further suggests a strong link between chronic short sleep and clinical issues, highlighting the value of early intervention. However, our results may not reflect the reality of chronic short sleepers with co‐occurring health issues, and future studies could broaden the criteria to include individuals with subclinical comorbidities or those whose subjective sleep need exceeds their habitual duration, exploring how this personalized approach can be adapted for more complex health contexts.

The large heterogeneity in barriers and strategy effectiveness argues for personalized interventions over one‐size‐fits‐all solutions. Context‐based modifications (e.g., phone distancing) proved more effective than simple behaviour substitution, supporting theoretical frameworks that emphasize environmental redesign over reliance on self‐control (Wood & Neal, 2007). Even for fixed environmental constraints, pragmatic adaptations such as reserving the bed strictly for sleep, in alignment with stimulus control principles, have shown promise (Bootzin et al., 1991).

Beyond physical environments, interventions should account for the biological sleep–wake rhythms and psychological drivers unique to young adulthood. Standard SH recommendations often fail this demographic because they overlook the biological tendency towards lateness which is further facilitated by students' high schedule autonomy. Rather than using this flexibility for sleep, students may use the late‐night hours to fulfil unmet psychological needs through media use (Jeoung et al., 2023). Such sleep delay behaviour can be understood as a way of coping with daytime stress or social needs, rather than simply as bad habits. Consequently, interventions should consider an individual's internal clock while simultaneously providing adaptive alternatives (e.g., healthier ways to decompress) to satisfy the underlying psychological drivers (Suh et al., 2022). By addressing both the biological timing and the psychological motivations for staying awake, practitioners can more effectively reduce the compulsion to delay sleep.

Moreover, an individual's cognitive and emotional profile may be critical for tailoring interventions. Individuals experiencing pre‐sleep arousal often find relief in strategies such as expressive journaling. Expressive writing may facilitate adjustment to stressors, and the effectiveness of the intervention for emotional outcomes may be partly linked to journaling mechanisms, as such practices have been shown to enhance adaptive ER and WB (Cheung et al., 2021). However, one participant resisted the practice, preferring to process emotions internally. Internal processing has been linked to less adaptive coping, potentially worsening sleep (Wang et al., 2024). Therefore, future interventions could be improved by assessing not only the barriers individuals face but also their typical coping style. Matching strategies to these styles could improve adherence; for example, individuals inclined towards internal processing may benefit more from guided mindfulness, which reduces pre‐sleep cognitive arousal (Hassirim et al., 2019), whereas those who benefit from externalization may prefer structured journaling.

Our results support comprehensive assessment and multiple analytics. Distinct patterns of change across different sleep parameters (duration, efficiency, fragmentation) highlight the necessity of measuring multiple sleep dimensions to fully understand intervention effects. Furthermore, employing both *t‐*tests/ANOVA and multilevel modelling proved valuable. Each method captured distinct aspects of the intervention, suggesting that both overall mean changes and nuanced within‐person dynamics are important to examine. Future studies should consider both trait‐level and state‐level processes when evaluating interventions. The consistent emergence of certain patterns under only specific analytic approaches argues for multimethod strategies rather than reliance on a single model.

### Limitations and main study design

While this pilot study suggested the feasibility of the personalized intervention, several limitations must be noted, which we attempt to address in an ongoing larger‐scale study (planned *N* = 225). In terms of the study design and intervention protocol, the pilot lacked a control group and a follow‐up phase, which precludes definitive causal conclusions about the specific effects of SE versus cumulative intervention exposure. Moreover, the 3‐day baseline was insufficient to fully capture natural sleep variations. The main study implements a controlled design with an SH‐only group and an extended protocol (comprising a one‐week baseline, one‐week SH, and two‐week combined phase), including a one‐month follow‐up. Recognizing that SE benefits may be transient (Ong et al., 2023), the main study incorporates specific behaviour change techniques, such as reflecting on successes and stimulating anticipation of future rewards (Hagger et al., 2014), to foster intrinsic motivation and a stronger sense of agency. Furthermore, to improve protocol adherence beyond mere data completion, the main study's incentive structure now links a portion of compensation directly to sleep behaviour change aims adherence.

Additionally, while the intensive, personalized contact in this pilot enhanced efficacy, such resource‐intensive procedures pose scalability challenges for clinical services. Existing research has begun exploring strategies to reduce clinician burden by shifting components to digital platforms (Baron et al., 2023) or group‐based formats (Chan et al., 2025). To address practical constraints, the main study will manualize the MI protocol to reduce dependency on specialized expertise, facilitating its potential integration into scalable digital health platforms.

Then, the small pilot sample size (*N* = 11) limited our statistical power to draw definitive conclusions from complex analyses. Although we employed multivariate modelling and two‐stage linear step‐up procedure to mitigate Type I error risks across multiple objectives, the stringency of these adjustments may have increased the risk of Type II errors for individual emotional dimensions. Consequently, these intervention findings on emotional outcomes remain exploratory and serve as analytic pathways for further investigation. The main study is sufficiently powered to formally test these psychological mechanisms (e.g., motivation and self‐efficacy) and identify for whom the intervention is most effective by examining baseline moderators.

Another limitation concerns the generalizability and participant context. Our rigorous screening and reliance on a student population may limit the generalizability of the findings. Also, students often enjoy particular affordances, such as flexible schedules, which differ from the rigid structural barriers (e.g., fixed work hours and family obligations) faced by working adults (Ong et al., 2023). To broaden the scope, our main study recruits from more diverse cohorts (including staff and part‐time workers). Additionally, the main study adds items on work/study day status and start time to the daily diary, which account for variations between weekdays and weekends that were not fully captured in this pilot. By recruiting a more diverse population and revising the daily diary to include these items, the main study will better capture varied life contexts and barriers to sleep improvement.

Methodologically, this pilot relied on subjective reports and basic actigraphy. To achieve a more robust mechanistic understanding, the main study incorporates polysomnography and additional wearables to capture sleep architecture (e.g., REM sleep) and link it to emotional outcomes. Behavioural tasks at pre‐ and post‐intervention will offer objective measures of emotional functioning. By using multilevel modelling to separate trait‐level effects from state‐level effects, the main study aims to advance from the preliminary associations established here towards a robust, mechanistic understanding of how extended sleep duration improves emotional health in chronic short sleepers.

## CONCLUSION

This pilot study showed that a personalized sleep intervention combining SH and SE is both feasible and effective for young adults with chronic short sleep. Although longer sleep was accompanied by reduced sleep efficiency, participants reported greater morning vitality. Emotional benefits were directly tied to the additional sleep gained each night. These mixed‐method findings suggest that effective sleep intervention should move beyond generalized advice to provide context‐based strategies concerning circadian rhythms, environmental constraints, and underlying psychological motivations and coping styles. While preliminary, the pilot results identify personalized intervention as a promising pathway to improve emotional health in chronically short sleepers, providing a foundation for future controlled trials to examine long‐term mechanisms and scalability.

## AUTHOR CONTRIBUTIONS

**Wei Wang:** Conceptualization; investigation; writing – original draft; methodology; formal analysis; project administration; writing – review and editing; data curation. **Ho Yin Derek Ma:** Investigation; writing – review and editing; visualization; data curation; writing – original draft. **Ming Ming Chiu:** Methodology; writing – review and editing; funding acquisition; supervision. **Shu Fai Cheung:** Methodology; writing – review and editing; supervision. **C. Harry Hui:** Writing – review and editing; supervision. **Mark Lawrence Wong:** Writing – review and editing; funding acquisition; supervision. **Janet Hui‐wen Hsiao:** Writing – review and editing; funding acquisition; supervision. **Rong Wei Sun:** Writing – review and editing; methodology; supervision. **Yeuk Ching Lam:** Funding acquisition; writing – review and editing; supervision. **Akira Basa Okabe:** Writing – review and editing. **Chin Ho Chan:** Writing – review and editing. **Esther Yuet Ying Lau:** Writing – review and editing; conceptualization; funding acquisition; supervision; project administration.

## CONFLICT OF INTEREST STATEMENT

The authors declare no conflict of interest.

## Supporting information


Data S1.

## Data Availability

The data that support the findings of this study are available from the corresponding author upon reasonable request.
