# Supplementary material for: From barriers to benefits: A personalized sleep intervention enhances sleep duration and emotional health in chronic short sleepers
Source: Br J Psychol. 2026 Apr 18;117(4):1469–94. doi: 10.1111/bjop.70075 (PMC13655946; doi:10.1111/bjop.70075)
Supplement: Supplementary file 1 [file BJOP-117-1469-s001.docx]

**Supplementary Figure 1.** *G*Power analysis*

*Note.* The achieved power was computed post hoc in G*Power 3.1. The analysis specified a "Means: Difference between two dependent means (matched pairs)" test. With a sample size of 11, a large effect size (dz = 0.90), and an α error probability of .05 (two-tailed), the result indicated a power of .77.

**Supplementary Figure 2.** *Monte Carlo simulations of primary outcomes*

**Supplementary Figure 3.** *Sleep duration changes across intervention stages*

*Note:* Each point represents the sleep duration of each participant for the corresponding day. Error bars represent the standard error of the mean. Smaller error bars mean a higher degree of consistency in actual sleep time among the 11 participants on that specific day.

**Supplementary Figure 4.** *Sleep duration by participant and intervention stage*

*Note:* Each point represents the sleep duration of the corresponding day.

**Supplementary Table 1.** *Detailed Participant Feedback Ratings (N=11)*

| Category | Item in Chinese | Item in English | Mean (SD) | Min | Max | % ≥ 4 |
| --- | --- | --- | --- | --- | --- | --- |
| Sleep Hygiene Component | 睡眠衛生規則很容易遵循 | Sleep hygiene rules are easy to follow | 3.91 (0.70) | 3 | 5 | 72.7 |
|  | 我獲得了有關睡眠衛生的知識 | I gained knowledge about sleep hygiene | 4.82 (0.40) | 4 | 5 | 100 |
|  | 實驗結束後，我會保持學習的習慣並繼續遵循睡眠衛生習慣 | After the experiment, I will maintain the habit of learning and continue to follow sleep hygiene practices | 4.73 (0.65) | 3 | 5 | 90.9 |
| Overall Experience | 我參加這個實驗是因為我有提高睡眠質量的意圖 | I participated in this experiment because I intend to improve my sleep quality | 4.55 (0.93) | 2 | 5 | 90.9 |
|  | 該實驗有助於改善我的睡眠質量 | This experiment helped improve my sleep quality | 4.73 (0.65) | 3 | 5 | 90.9 |
|  | 總的來說，我對整個實驗感到滿意 | Overall, I am satisfied with the entire experiment | 4.55 (0.69) | 3 | 5 | 90.9 |
|  | 如果有機會再做一次類似的實驗，我很樂意參與 | I would be willing to participate in a similar experiment again if given the opportunity | 4.18 (1.33) | 1 | 5 | 72.7 |
| Sleep Extension Component | 我很容易將睡眠時間延長 90 分鐘 | I find it easy to extend my sleep time by 90 minutes | 3.64 (0.92) | 2 | 5 | 54.5 |
|  | 我覺得延長睡眠時間 90 分鐘很舒服 | I feel comfortable extending my sleep time by 90 minutes | 4.64 (0.50) | 4 | 5 | 100 |
|  | 我覺得延長睡眠時間 90 分鐘有助於提高我的認知功能，包括記憶力，計劃能力，警覺性等 | I believe extending my sleep time by 90 minutes helps improve my cognitive functions, including memory, planning, and alertness | 4.82 (0.40) | 4 | 5 | 100 |
|  | 我覺得延長睡眠時間 90 分鐘對我情緒的改善有幫助 | I believe extending my sleep time by 90 minutes helps improve my mood | 4.91 (0.30) | 4 | 5 | 100 |
|  | 我很難將睡眠時間延長 90 分鐘 | I find it difficult to extend my sleep time by 90 minutes | 1.82 (0.98) | 1 | 4 | 54.5 |
|  | 實驗結束後，我會繼續延長我的睡眠時間 | After the experiment, I will continue to extend my sleep time | 4.73 (0.47) | 4 | 5 | 100 |
| Methodological Aspects | 在整個實驗期間，我覺得佩戴活動記錄手表很舒服 | During the entire experiment, I found wearing the activity monitor comfortable | 3.82 (0.98) | 2 | 5 | 81.8 |
|  | 在實驗室睡覺的晚上，佩戴儀器不會影響我的睡眠 | Wearing the device does not affect my sleep on the nights I sleep in the lab | 3.00 (1.67) | 1 | 5 | 45.5 |
|  | 早晚填寫的睡眠日記方便使用且易於完成 | The sleep diary completed morning and evening is convenient and easy to fill out | 4.00 (0.77) | 2 | 5 | 90.9 |
|  | 四輪問卷都很容易完成 | All four questionnaires are easy to complete | 3.64 (0.92) | 2 | 5 | 54.5 |
|  | 研究人員提供了充分的支持和明確的指示 | The researchers provided ample support and clear instructions | 4.64 (0.67) | 3 | 5 | 90.9 |
|  | 我很滿意研究補償的金額 | I am satisfied with the amount of compensation for the study | 3.73 (1.56) | 1 | 5 | 63.6 |
| Open-ended question | 您對我們的研究還有其他反饋嗎？ | Do you have any other feedback on the research study? | (1 response received) The researchers had a great attitude and were very patient. Through the experiment, I found a method that works for me to increase my sleep time. | | | |

*Note:* Rating for all items range from 1-5 (very disagree; disagree; not sure; agree; very agree).

**Supplementary Table 2a.** *Interview questions and procedures for the semi-structured MI-based sleep hygiene interview at T2.*

| Section | Objective | MI module | Details | Example semi-structured questions |
| --- | --- | --- | --- | --- |
| Introduction | Outlining the session's collaborative nature and providing an overview of the session | Engaging | To introduce the focus of improving SH and sleep quality and engaging the participants | a) “What do you know about your sleep?” |
| Review | Exploring the participant's personal experience and understanding of their habitual sleep pattern (T1-T2) | Engaging + Focusing | a) review actigraphy motionlogging data and daily diary self-report to help participants understand their daytime habits and sleep characteristics (especially sleep variability)  b) enquire participants' subjective feelings and thoughts regarding their own sleep | a) "How do you feel about your current sleep patterns?"  b) "What are some reasons that could explain your current sleep pattern?" |
| Education (1 of 2) | Providing sleep hygiene knowledge and introducing Wendy Wood's (2019) conceptualisation of habit building, and invites curiosity and connects to the participant's experience | - | a) exploring the habitual nature of sleep hygiene  b) understanding how habits are built through promoting behavioural automaticity  c) listing common outcomes of poor sleep hygiene (e.g., insomnia) and maladaptive habits (e.g., media use close to bedtime and in bed) | a) "The idea of 'habits' is that they run on autopilot. Does that resonate with any part of your evening routine?" |
| Reflection | Inviting participants to fill in questionnaires regarding their sleep habits and barriers | Focusing + Evoking | a) participants to fill in the Integrated Scale for Sleep hygiene practices and the scale for Perceived Barriers to Sufficient Sleep  b) reviewing the participants' choices to each item and evaluate specific items pertinent to the participants' situation, to enquire further on the thoughts and perception in regard to sleep hygiene  c) examine prominent barriers that participants are interested in modifying and setting aims to address said barriers | a) "It appears you engage in media use 30 minutes before bed almost every day in the past week, may I know the details?"  b) "If you could change one thing about your pre-sleep time, what would have the biggest positive impact for you?"  c) "What are some things you've already tried, and what did you learn from that?" |
| Education (2 of 2) | sleep hygiene video watching - common adaptive sleep hygiene practices to be shared to participants | Evoking | Providing examples of sleep hygiene practices (e.g., earlier caffeine use to preserve sleepiness at night; adjusting social media use before sleep) | a) "Would it be okay if I shared a couple of common strategies people use?"  b) "What are your thoughts on these? Does any of that sound like it might fit for you?" |
| Addressing barriers | Collaboratively building a plan based on the participant's own ideas and motivation. | Planning | a) revisiting Wendy Wood's (2019) conceptualisation of habit building  b) helping participants develop their tailored SH behaviour change plan through nudges, cues, and conditioning through inducing behavioural automaticity across T2-T3  c) assist in solidifying participants' plans (i.e., what is the specific desired behaviour; when, where, and how to carry out said behaviour) | a) "Given everything we've discussed, what, if anything, feels like a next step you'd be willing to try?"  b) "You mentioned wanting to feel more relaxed before bed. What could a small first step toward that look like?"  c) “What would help you to be successful in achieving your goals?” |
| Conclusion | Summarizing the participant's own words and reinforcing their autonomy | - | a) providing a concise summary of the concepts visited and the barriers to-be addressed  b) scheduling date and time for the next interview at T3  c) completing multiple scales to assess the participants' motivation and self-efficacy in carrying out their plans | "Let me see if I understand what you've shared. You want to X, but Y is impeding you, and you're considering trying Z as a step toward that. Did I get that right?"  "It's completely up to you what you do with this. What are your final thoughts before we finish up today?" |

**Supplementary Table 2b.** *Interview questions and procedures for the semi-structured MI-based sleep hygiene interview at T3.*

| Section | Objective | MI module | Details | Example semi-structured questions |
| --- | --- | --- | --- | --- |
| Introduction | Outlining the integrated sleep hygiene and extension intervention and providing an overview of the session | Engaging | To introduce the focus of reviewing SH across T2-T3, and to help design a personal sleep extension plan | - |
| Review | Exploring participants' personal experiences and gains during sleep hygiene phase (T2-T3) | Engaging + Focusing | a) review actigraphy motionlogging data and daily diary self-report to help participants visualise changes in their daytime habits and sleep charateristics (T2-T3) compared to the baseline period (T1-T2)  b) enquire participants' subjective feelings and thoughts regarding their changes, along with any developments in their sleep habits  c) providing feedback and encouragment to participants' successes and examining difficulties or determinants to the success of their plan | a) “What do you think has changed about your sleep in the past few days?”  b) "How has the sleep hygiene targets helped you sleep better?"  c) "Have you encountered any difficulties with adjusting to your new sleep targets?" |
| Education | Providing empirical information on the risks of chronic short sleep | Evoking | a) exploring the empirical associations and outcomes of chronic short sleep (e.g., relationship between short sleep and depression/ anxiety, emotional reactivity)  b) exploring the empirically backed findings of extended sleep duration | - |
| Sleep Extension | Collaboratively setting a personalised 90-minute sleep extension plan across T3-T4 | Planning | a) according to the daily schedules of participants, to design a personalised 90-minute SE plan (e.g., going to bed 90 minutes earlier; going to bed 30 minutes earlier and getting out of bed 60 minutes later)  b) acknowledging the difficulty of immediately extending such a long period of sleep, and suggest a gradual increase of sleep duration across the first three days after the intervention | a) "Since you want to sleep more, how do you plan on achieving that?"  b) "How would you like to sleep longer? How should we schedule your sleep and wake times?"  c) "What are some of the potential difficulties for you to extend your sleep?" |
| Conclusion | Summarise the contents of the interview and assessing change motivation | - | a) providing a concise summary of the concepts visited and the SE plan  b) completing multiple scales to assess the participants' motivation and self-efficacy in carrying out their plans |  |

**Supplementary Table 3.** *The exact number of days completed per participant in each stage*

| ID | Baseline | Sleep hygiene | Sleep extension |
| --- | --- | --- | --- |
| 01 | 4 | 4 | 7 |
| 02 | 3 | 5 | 6 |
| 03 | 7 | 4 | 11 |
| 04 | 11 | 4 | 10 |
| 05 | 3 | 5 | 6 |
| 06 | 3 | 5 | 7 |
| 07 | 3 | 5 | 7 |
| 08 | 5 | 3 | 6 |
| 09 | 3 | 4 | 7 |
| 10 | 3 | 5 | 7 |
| 11 | 3 | 4 | 7 |

**Supplementary Table 4.** *Multilevel variable variance*

| Variable | ID_Var | Daily_Var | Moment_Var |
| --- | --- | --- | --- |
| Wellbeing | 0.596 | 0.404 | NA |
| Positive Affect | 0.646 | 0.354 | NA |
| Negative Affect | 0.251 | 0.749 | NA |
| Total Sleep Time | 0.52 | 0.48 | NA |
| Sleep Efficiency | 0.152 | 0.848 | NA |
| Sleep Latency | 0.322 | 0.678 | NA |
| Fragmentation Index | 0.012 | 0.988 | NA |
| Sleep Midpoint | 0.307 | 0.693 | NA |
| Sleep Quality | 0.525 | 0.475 | NA |
| Acceptance | 0.352 | 0.125 | 0.522 |
| Distraction | 0.65 | 0.031 | 0.318 |
| Avoidance | 0.735 | 0.05 | 0.215 |
| Suppression | 0.676 | 0.04 | 0.284 |
| Reappraisal | 0.564 | 0.047 | 0.388 |
| Rumination | 0.601 | 0.065 | 0.334 |

*Note.* Variance components were estimated by fitting unconditional means models (intercept-only models) using linear mixed-effects modelling. For daily-level variables (assessed once per day), a two-level model was specified with observations nested within participants (Variable ~ 1 + (1 | ID)). The variance was partitioned into a between-person component (ID_Var; ICC) and a within-person residual component (Daily_Var). For Ecological Momentary Assessment (EMA) variables (assessed multiple times per day), a three-level model was specified with moments nested within days, nested within participants (Variable ~ 1 + (1 | ID) + (1 | ID:Day)). The variance was partitioned into a between-person component (ID_Var), a day-to-day within-person component (Day_Var), and a momentary within-person residual component (Moment_Var). All values represent the proportion of total variance accounted for by each level.

**Supplementary Table 5a.** *Application of the Two-Stage Linear Step-Up Procedure for False Discovery Rate (FDR) Control in Moderator Analyses*

Given the pilot nature of this study and the restricted sample size (N = 11), the following moderation analyses were conducted for exploratory purposes and hypothesis generation for the subsequent main study. These results should be interpreted with extreme caution due to low statistical power and the inherent risk of spurious correlations.

Exploratory analyses tested whether baseline variables moderated the effect of SE on TST. The uncorrected results showed three moderators: barrier reduction (*β* = 0.098, *p* = .009), emotional stability (*β* = −0.099, *p* = .011), and baseline sleep duration (*β* = 0.783, *p* = .002). However, none of these interactions survived a two-stage linear step-up procedure for false discovery rate control (refer to ST 4a, 4b for procedures). Given the pilot nature of this study, we conducted exploratory simple slope analyses to probe these patterns for future hypothesis generation. The results suggested that the effect of SE on TST was more pronounced for individuals with longer baseline sleep duration (+0.94 hours), lower emotion stability (+0.90 hours), and those who solved more barriers from interviews (+1.14 hours, ST 5 for details).

Note that the direction and magnitude of these preliminary slopes may be disproportionately influenced by individual data points within this small sample (N=11), leading to potentially noisy or counterintuitive trends. These patterns are considered preliminary and will be formally tested in the adequately powered main study.

| Rank | Term | Estimate | Std. Error | Statistic | DF | P Value |
| --- | --- | --- | --- | --- | --- | --- |
| 1 | Barrier Improvement | 0.098 | 0.037 | 2.641 | 156.225 | 0.009 |
| 2 | Emotion Stability | -0.088 | 0.034 | -2.586 | 166.814 | 0.011 |
| 3 | Baseline Sleep Duration | 0.783 | 0.341 | 2.297 | 159.018 | 0.023 |
| 4 | Conscientiousness | 0.058 | 0.033 | 1.729 | 157.182 | 0.086 |
| 5 | Baseline Sleep Quality | -0.151 | 0.096 | -1.581 | 166.648 | 0.116 |
| 6 | Age | -0.175 | 0.111 | -1.572 | 166.267 | 0.118 |
| 7 | Bed Procrastination | -0.048 | 0.036 | -1.356 | 160.046 | 0.177 |
| 8 | Sleep Debt | -0.267 | 0.235 | -1.136 | 163.536 | 0.257 |
| 9 | Motivation and Attitude to Change | 0.052 | 0.051 | 1.021 | 159.835 | 0.309 |
| 10 | Sleep Hygiene Practice | -0.015 | 0.017 | -0.866 | 167.018 | 0.388 |
| 11 | Chronic Sleep Reduction | 0.008 | 0.028 | 0.279 | 165.693 | 0.781 |
| 12 | Self-Sleep Efficiency | -0.009 | 0.035 | -0.259 | 162.629 | 0.796 |
| 13 | Self-Regulation | -0.008 | 0.035 | -0.222 | 166.573 | 0.825 |

*Note.* This procedure controls the False Discovery Rate at a = .05. The total number of hypotheses tested (T) is 13. The adjusted significance threshold for the first stage is calculated as follows:

1) a’ = .05 / (1 + .05) = .048

2) Sorted p-values from the regression coefficients and standard errors

3) We computed the steps of a'

T = total # of hypotheses = 12

r = current number of rejected hypotheses = 0

u = number of remaining unrejected hypotheses = 13

For i = 1… u, Compute a’ x i / u

4) Test each pi against a’ x i / u

**Supplementary Table 5b.** *Follow-up explanatory variable testing*

| i | Explanatory variable | *p* | a’ x i / u | Reject? |
| --- | --- | --- | --- | --- |
| 1 | Barrier Improvement | 0.009 | 0.004 | N |
| 2 | Emotion Stability | 0.011 | 0.007 | N |
| 3 | Baseline Sleep Duration | 0.023 | 0.011 | N |
| 4 | Conscientiousness | 0.086 | 0.015 | N |
| 5 | Baseline Sleep Quality | 0.116 | 0.018 | N |
| 6 | Age | 0.118 | 0.022 | N |
| 7 | Bed Procrastination | 0.177 | 0.026 | N |
| 8 | Sleep Debt | 0.257 | 0.030 | N |
| 9 | Motivation and Attitude to Change | 0.309 | 0.033 | N |
| 10 | Sleep Hygiene Practice | 0.388 | 0.037 | N |
| 11 | Chronic Sleep Reduction | 0.781 | 0.041 | N |
| 12 | Self-Sleep Efficiency | 0.796 | 0.044 | N |
| 13 | Self-Regulation | 0.825 | 0.048 | N |

*Note:* 5) No significant moderators were identified after FDR correction.

**Supplementary Table 6.** *Emotion changes from baseline (T2) to post-sleep extension (T4).*

| Variable | Mean (SD) | | Paired t-test | | | 95% CI | |
| --- | --- | --- | --- | --- | --- | --- | --- |
|  | Pre | Post | t (df) | Two-sided *p* | Cohen’s d | Lower | Upper |
| DASS - Depression | 1.55 (0.69) | 1.36 (0.67) | 0.56 (10) | 0.59 | 0.17 | -0.54 | 0.91 |
| DASS - Anxiety | 1.00 (0.00) | 1.18 (0.40) | -1.42 (10) | 0.19 | 0.43 | -0.45 | 0.09 |
| DASS - Stress | 1.55 (0.82) | 1.55 (0.69) | 0.92 (10) | 0.38 | 0.28 | -0.67 | 0.67 |
| PANAS - Positive affect | 26.73 (4.31) | 26.45 (3.88) | 0.16 (10) | 0.88 | 0.05 | -3.61 | 4.16 |
| PANAS - Negative emotion | 21.18 (4.26) | 20.91 (3.67) | 0.21 (10) | 0.84 | 0.06 | -2.64 | 3.19 |
| Positive emotional reactivity | 34.91 (4.06) | 33.91 (4.32) | 0.57 (10) | 0.58 | 0.17 | -5.26 | 4.53 |
| Negative emotional reactivity | 23.09 (6.16) | 23.45 (9.16) | -0.17 (10) | 0.87 | 0.05 | -2.88 | 4.88 |
| ERQ - Cognitive reappraisal | 29.64 (5.71) | 30.91 (6.92) | -0.57 (10) | 0.58 | 0.17 | -6.25 | 3.70 |
| ERQ - Expressive suppression | 12.27 (4.56) | 12.45 (5.50) | -0.11 (10) | 0.92 | 0.03 | -3.92 | 3.56 |

**Supplementary Table7.** *Multilevel Mediation Analysis Results: Sleep Duration, Sleep Quality, and Emotional Outcomes*

| Outcome | Model Type | Path | Estimate | 95% CI | Significant |
| --- | --- | --- | --- | --- | --- |
| Well-Being | Absolute Levels | a (Duration→Quality) | 0.188 | [0.06, 0.316] | Yes |
|  |  | b (Quality→WB) | 0.755 | [-0.283, 1.793] | No |
|  |  | Indirect (a×b) | 0.157 | [-0.084, 0.397] | No |
|  |  | Direct (Duration→WB) | 0.43 | [-0.082, 0.943] | No |
|  | Gain Scores | a (Gain→Quality Gain) | 0.201 | [0.058, 0.343] | Yes |
|  |  | b (Quality Gain→WB) | 0.84 | [-0.598, 2.279] | No |
|  |  | Indirect (a×b) | 0.184 | [-0.203, 0.571] | No |
|  |  | Direct (Gain→WB) | 0.336 | [-0.218, 0.891] | No |
| Positive Affect | Absolute Levels | a (Duration→Quality) | 0.195 | [0.065, 0.325] | Yes |
|  |  | b (Quality→PA) | 0.107 | [-0.739, 0.953] | No |
|  |  | Indirect (a×b) | 0.026 | [-0.166, 0.219] | No |
|  |  | Direct (Duration→PA) | 0.452 | [0.062, 0.843] | Yes |
|  | Gain Scores | a (Gain→Quality Gain) | 0.2 | [0.054, 0.345] | Yes |
|  |  | b (Quality Gain→PA) | 0.171 | [-0.982, 1.324] | No |
|  |  | Indirect (a×b) | 0.031 | [-0.289, 0.35] | No |
|  |  | Direct (Gain→PA) | 0.433 | [0.03, 0.837] | Yes |
| Negative Affect | Absolute Levels | a (Duration→Quality) | 0.188 | [0.061, 0.315] | Yes |
|  |  | b (Quality→NA) | -0.504 | [-1.161, 0.154] | No |
|  |  | Indirect (a×b) | -0.099 | [-0.253, 0.055] | No |
|  |  | Direct (Duration→NA) | -0.25 | [-0.626, 0.126] | No |
|  | Gain Scores | a (Gain→Quality Gain) | 0.206 | [0.061, 0.351] | Yes |
|  |  | b (Quality Gain→NA) | -0.497 | [-1.249, 0.254] | No |
|  |  | Indirect (a×b) | -0.107 | [-0.316, 0.101] | No |
|  |  | Direct (Gain→NA) | -0.221 | [-0.607, 0.166] | No |
| Rumination | Absolute Levels | a (Duration→Quality) | 0.191 | [0.062, 0.321] | Yes |
|  |  | b (Quality→Rumination) | -0.114 | [-0.291, 0.062] | No |
|  |  | Indirect (a×b) | -0.024 | [-0.066, 0.019] | No |
|  |  | Direct (Duration→Rumination) | -0.11 | [-0.184, -0.036] | Yes |
|  | Gain Scores | a (Gain→Quality Gain) | 0.197 | [0.055, 0.339] | Yes |
|  |  | b (Quality Gain→Rumination) | -0.114 | [-0.324, 0.096] | No |
|  |  | Indirect (a×b) | -0.022 | [-0.079, 0.035] | No |
|  |  | Direct (Gain→Rumination) | -0.109 | [-0.183, -0.035] | Yes |
| Acceptance | Absolute Levels | a (Duration→Quality) | 0.188 | [0.058, 0.317] | Yes |
|  |  | b (Quality→ER_acc) | -0.035 | [-0.217, 0.147] | No |
|  |  | Indirect (a×b) | -0.009 | [-0.050, 0.032] | No |
|  |  | c' (Direct) | 0.077 | [-0.009, 0.164] | No |
|  | Gain Scores | a (Duration→Quality) | 0.195 | [0.047, 0.343] | Yes |
|  |  | b (Quality→ER_acc) | -0.027 | [-0.270, 0.216] | No |
|  |  | Indirect (a×b) | -0.009 | [-0.077, 0.059] | No |
|  |  | c' (Direct) | 0.085 | [-0.003, 0.174] | No |
| Distraction | Absolute Levels | a (Duration→Quality) | 0.192 | [0.063, 0.321] | Yes |
|  |  | b (Quality→distraction) | 0.045 | [-0.103, 0.194] | No |
|  |  | Indirect (a×b) | 0.009 | [-0.025, 0.043] | No |
|  |  | c' (Direct) | 0.059 | [-0.019, 0.137] | No |
|  | Gain Scores | a (Duration→Quality) | 0.196 | [0.046, 0.345] | Yes |
|  |  | b (Quality→distraction) | 0.042 | [-0.107, 0.191] | No |
|  |  | Indirect (a×b) | 0.007 | [-0.031, 0.046] | No |
|  |  | c' (Direct) | 0.063 | [-0.016, 0.142] | No |
| Avoidance | Absolute Levels | a (Duration→Quality) | 0.189 | [0.059, 0.319] | Yes |
|  |  | b (Quality→avoidance) | -0.176 | [-0.442, 0.090] | No |
|  |  | Indirect (a×b) | -0.033 | [-0.096, 0.030] | No |
|  |  | c' (Direct) | -0.021 | [-0.103, 0.061] | No |
|  | Gain Scores | a (Duration→Quality) | 0.193 | [0.045, 0.342] | Yes |
|  |  | b (Quality→avoidance) | -0.19 | [-0.477, 0.097] | No |
|  |  | Indirect (a×b) | -0.036 | [-0.114, 0.042] | No |
|  |  | c' (Direct) | -0.014 | [-0.097, 0.070] | No |
| Suppression | Absolute Levels | a (Duration→Quality) | 0.191 | [0.061, 0.322] | Yes |
|  |  | b (Quality→suppression) | -0.003 | [-0.190, 0.185] | No |
|  |  | Indirect (a×b) | 0.001 | [-0.039, 0.042] | No |
|  |  | c' (Direct) | -0.028 | [-0.106, 0.051] | No |
|  | Gain Scores | a (Duration→Quality) | 0.197 | [0.047, 0.347] | Yes |
|  |  | b (Quality→suppression) | -0.005 | [-0.197, 0.187] | No |
|  |  | Indirect (a×b) | 0.002 | [-0.046, 0.050] | No |
|  |  | c' (Direct) | -0.023 | [-0.102, 0.056] | No |
| Reappraisal | Absolute Levels | a (Duration→Quality) | 0.188 | [0.056, 0.319] | Yes |
|  |  | b (Quality→reappraisal) | -0.032 | [-0.178, 0.114] | No |
|  |  | Indirect (a×b) | -0.007 | [-0.039, 0.025] | No |
|  |  | c' (Direct) | 0.106 | [0.024, 0.188] | Yes |
|  | Gain Scores | a (Duration→Quality) | 0.19 | [0.033, 0.348] | Yes |
|  |  | b (Quality→reappraisal) | -0.031 | [-0.185, 0.123] | No |
|  |  | Indirect (a×b) | -0.007 | [-0.046, 0.032] | No |
|  |  | c' (Direct) | 0.108 | [0.025, 0.190] | Yes |

*Note:* All models were run on intervention phase data only. Absolute Levels models use daily raw scores; Gain Scores models use daily deviations from individual baseline means.
